# Supplementary material for: Maintenance of pluripotency-like signature in the entire ectoderm leads to neural crest stem cell potential
Source: Nat Commun. 2023 Sep 23;14:5941. doi: 10.1038/s41467-023-41384-6 (PMC10518019; doi:10.1038/s41467-023-41384-6)
Supplement: Supplementary file 1 — Supplementary Information File [file 41467_2023_41384_MOESM1_ESM.pdf]

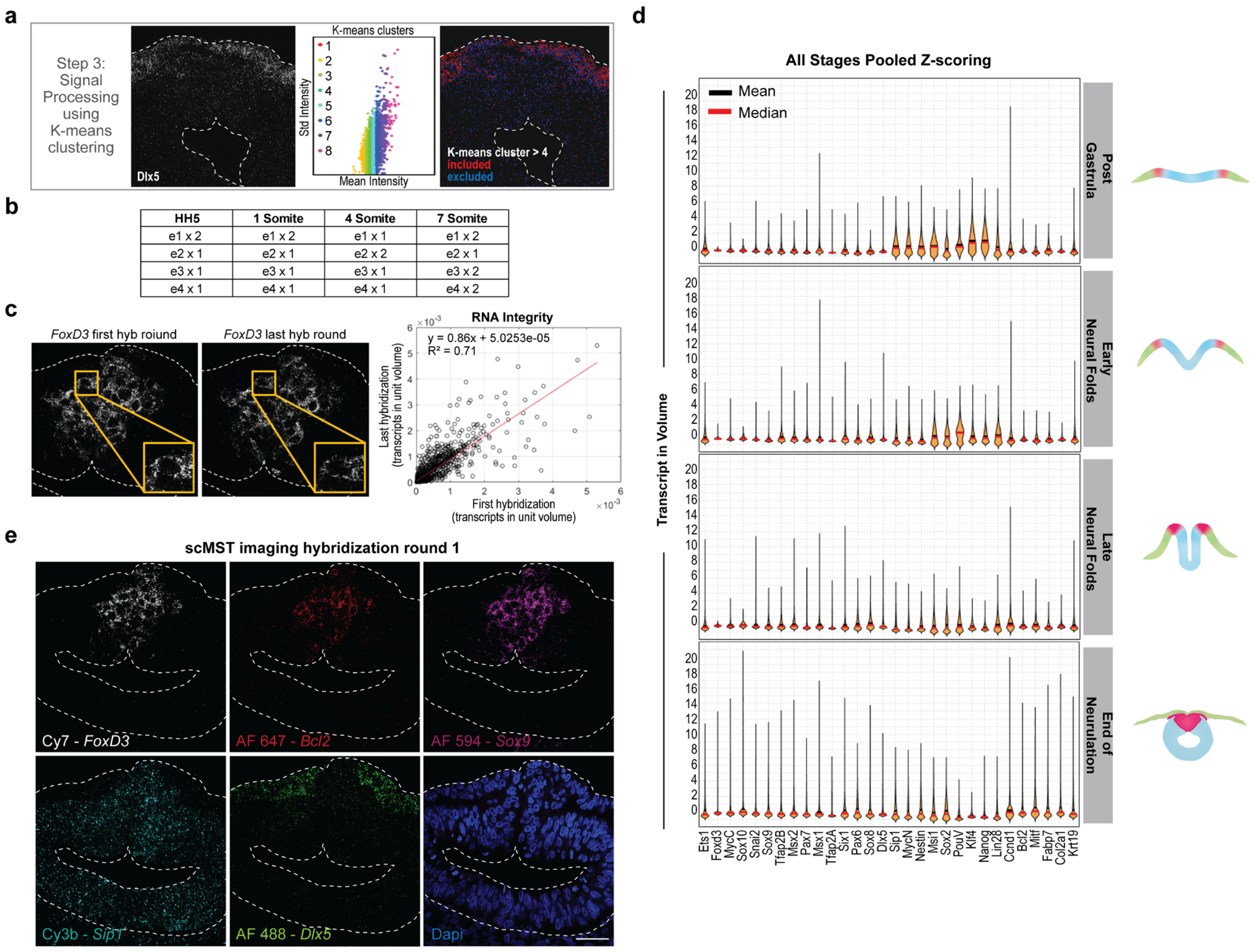

Supplementary Figure 1 | Single cell multiplex spatial transcriptomics (scMST) signal processing and gene expression validation.

a, K-means clustering in the signal processing step of the scMST pipeline identifies dots (RNA transcripts) with similar signal intensities, enabling unbiased inclusion of specific and exclusion of unspecific signal from downstream analysis. Example image shows signal processing step for the expression of the *Dlx5* gene where clusters 5- 8 are chosen to be included as true signal (red) and rest of the clusters are excluded as background signal (blue). **b**, The table displays the number of sections per embryo that were pooled for each stage included in the scMST analysis. **c**, RNA integrity is preserved after nine rounds of re-hybridization, with 71% of transcripts remaining intact. Comparison of raw transcript images and correlation plot for *FoxD3* shown as a representative of the success of RNA integrity maintenance during scMST rounds. **d**, Violin plots were generated to enable gene expression comparison across multiple stages, shown as transcripts in volume, which are normalized among genes and samples. Note that the data includes pooled cells from all germ layers within the midbrain level tissue. **e**, Example of an imaging round in our scMST method (Hyb1, 7ss sample). The

figure displays five different RNA channels and one DAPI channel to demonstrate the absence of any cross-talk or leakage between channels. Scale bar = 30 μm.

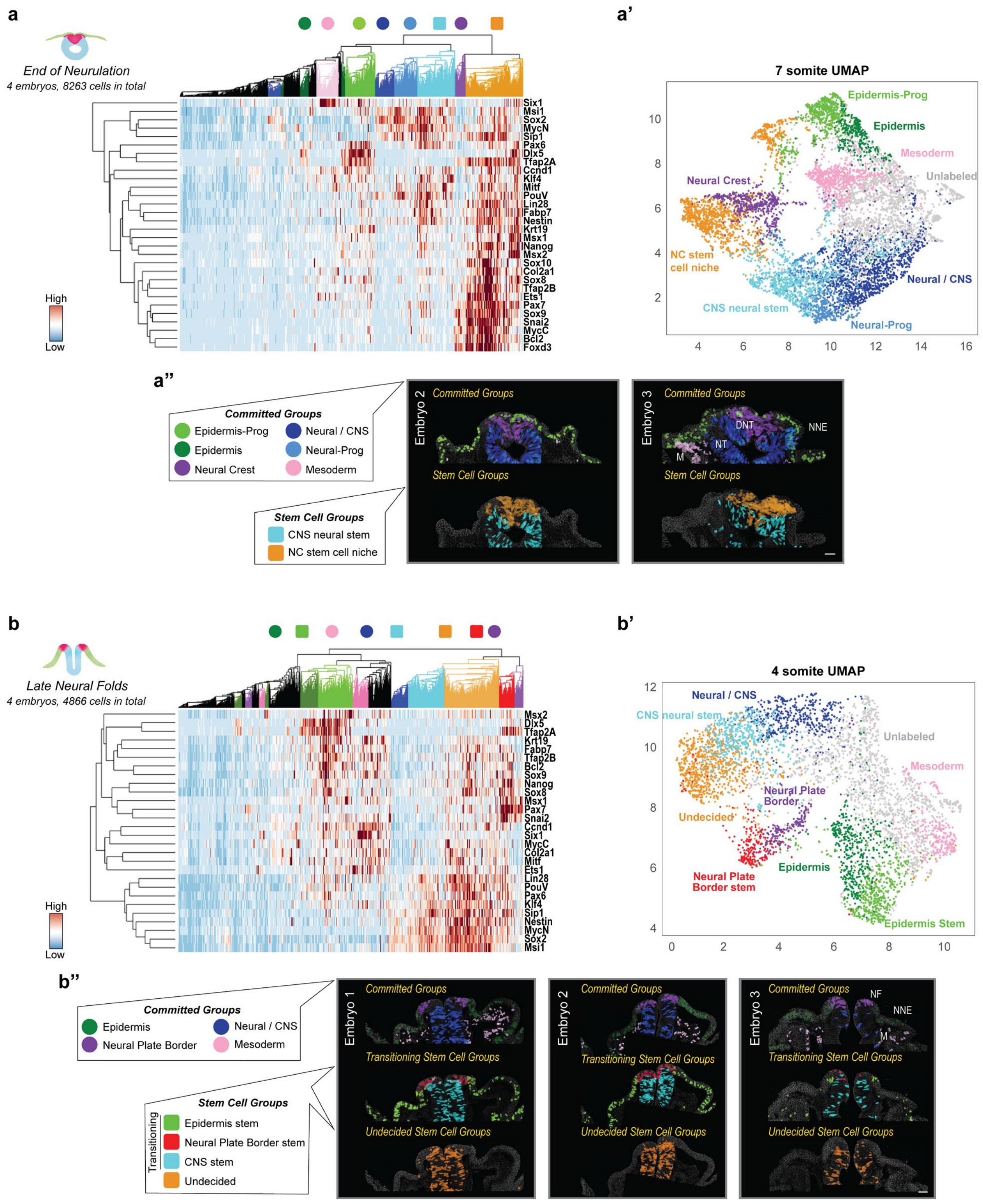

Supplementary Figure 2 | scMST results shown as pooled heatmaps from parallel samples together with pseudo-colored embryo images (7ss and 4ss)

a, scMST heatmap for end of neurulation stage (7ss, HH9, the endpoint of our study) highlights transcriptionally distinct subpopulations using a color-coding scheme. **a'**, The same identified subpopulations displayed in a UMAP. **a''**, Two additional pseudo-colored embryos represent the

spatial location of cells representing each respective subpopulation of the pooled heatmap, replicating the spatial pattern shown in figure 1d. **b**, scMST heatmap for late neural fold stage (4ss, HH7) shows transcriptionally distinct committed populations that do not express pluripotency genes or markers of the neighboring domains. The transcriptional profile of all the respective transitioning stem cell populations suggests that the future fate is already dictated based on stronger expression of genes of one domain, while the cells also still express genes of the neighboring domains together with pluripotency genes, indicating an intermediate persistence of plasticity. The undecided group co-express pluripotency genes and shares expression of neural and NC markers indicating the highest level of stemness in the ectoderm. **b'**, The same identified subpopulations displayed in a UMAP. **b''**, Spatial back-mapping of cells in respective subgroups from the pooled heatmap into the original tissue images from three different embryos demonstrates reproducibility of the spatial patterning of the transcriptionally distinct subpopulations. Pseudo-colored cells show that while committed cell groups do not overlap with each other's domains, the transitioning stem cell groups overlap with other domains at the borders, and the undecided stem cells predominantly span over neural and neural crest domains. Source data for scMST are provided as a Source Data file. NNE=Non-Neural Ectoderm, NF=Neural Fold, M=Mesoderm, Scale bar = 30 μm

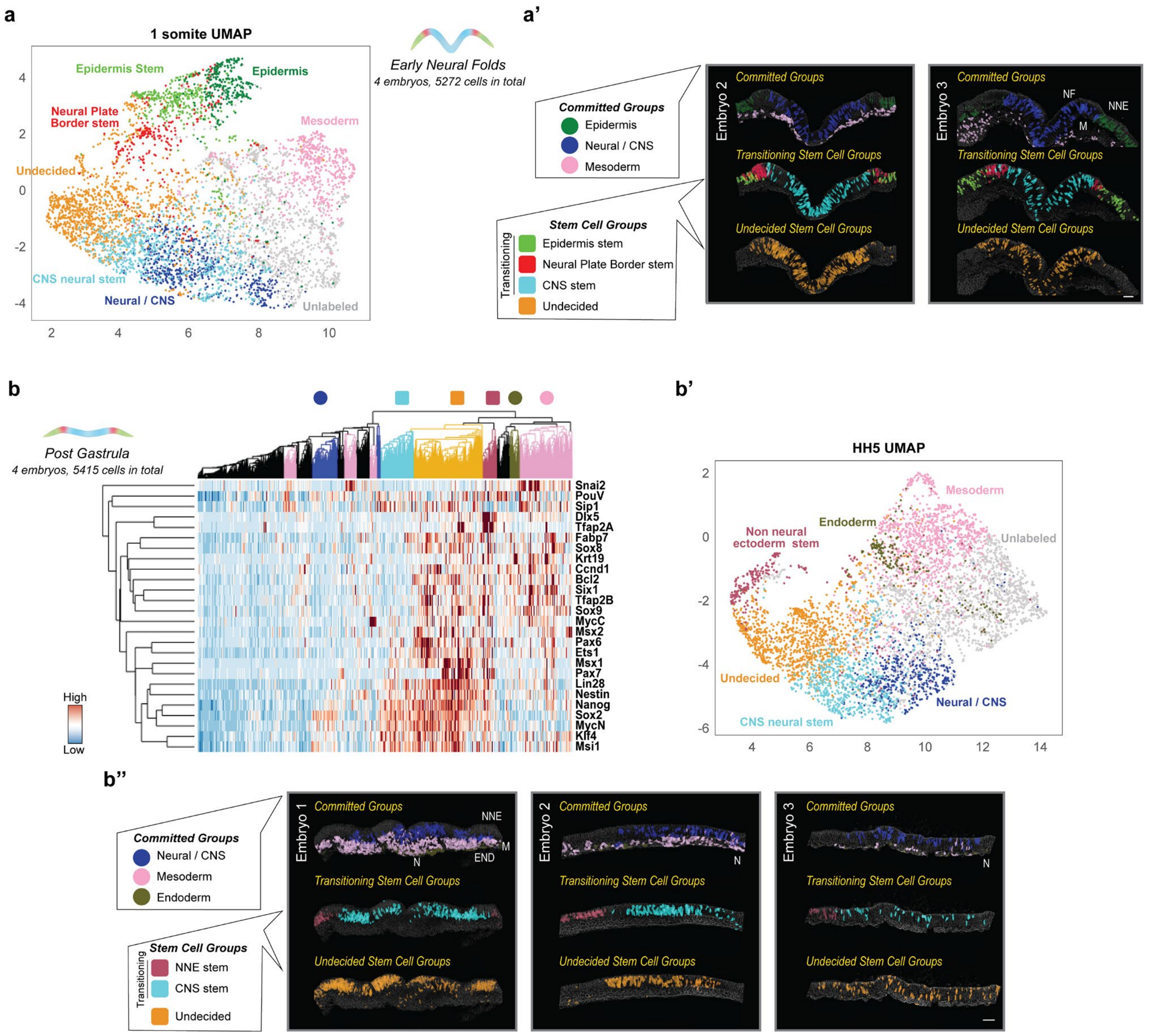

Supplementary Figure 3 | scMST results shown as pooled heatmaps and the spatial distribution of transcriptionally distinct subdomains from parallel samples (1ss and HH5)

a, The same identified subpopulations from the pooled heatmap in Fig. 1e displayed in a UMAP. **a'**, Spatial distribution of cells in the subpopulations of the pooled heatmap in Fig 1e in two additional embryos demonstrates reproducibility of the spatial pattern between embryos. **b**, scMST heatmap for gastrula stage (HH5) shows ectodermal subpopulations mostly consist of undecided stem cell groups, although some committed cells of future CNS, together with mesoderm and endoderm are detectable. **b'** The same identified subpopulations from the pooled heatmap displayed in a UMAP. **b''**, Visualization of the subpopulations show spatial location of the committed subpopulations, while the majority of the ectodermal cells are undecided stem cells that span the entire ectoderm. Note that the notochord (N) is in the center of embryo 1, and on the side in embryos 2-3 to capture the very lateral parts of the ectoderm. Source data for scMST are provided as a Source Data file. HH= Hamburger

Hamilton chicken stage, NNE=Non-Neural Ectoderm, NF=Neural Fold, M=Mesoderm, END=Endoderm, N=Notochord (midline), Scale bar = 30 μm.

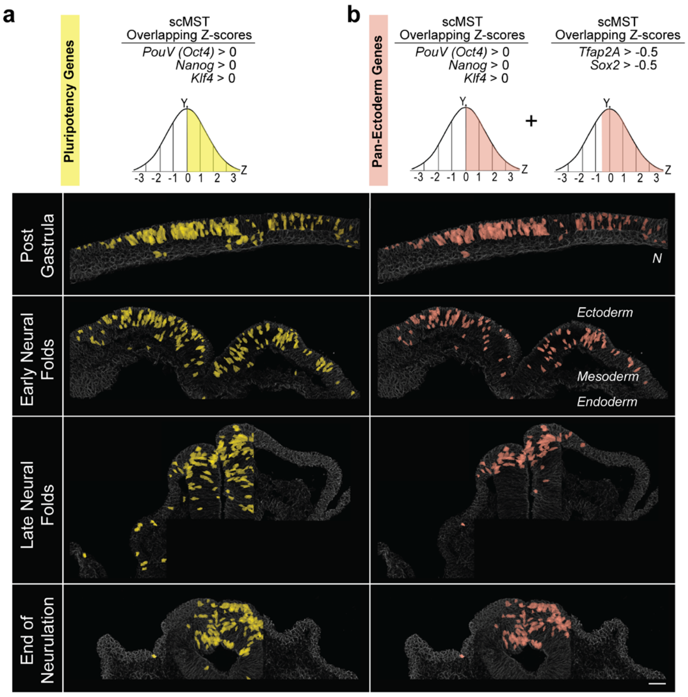

c

Stages	Total cell count per stage	Pluri positive (yellow) cells	Pan-Ecto (pink) cells	Pluri cells overlapping with Pan-Ecto	percentage
HH5	5415	919	793	793	86.30 %
1 somite	5272	1010	688	688	68.10 %
4 somite	4866	771	374	374	48.50 %
7 somite	8263	965	711	711	73.70 %
				average percentage =	69,1%

d

Stage	Stem cell Groups from heatmap	number of cells	Pan-Ecto stem cells	number of overlapping cells with Pan-Ecto	percentage
HH5	Transitioning	889		238	
	Undecided	1179		457	
	Both groups together	2068	793	689	87%
1 somite	Transitioning	1436		181	
	Undecided	1228		478	
	Both groups together	2664	688	659	96%
4 somite	Transitioning	1241		177	
	Undecided	804		181	
	Both groups together	2045	374	358	96%
7 somite	Nstem	1066		104	
	NC stem niche	1251		538	
	Both groups together	2317	711	642	90%
				average percentage =	92%

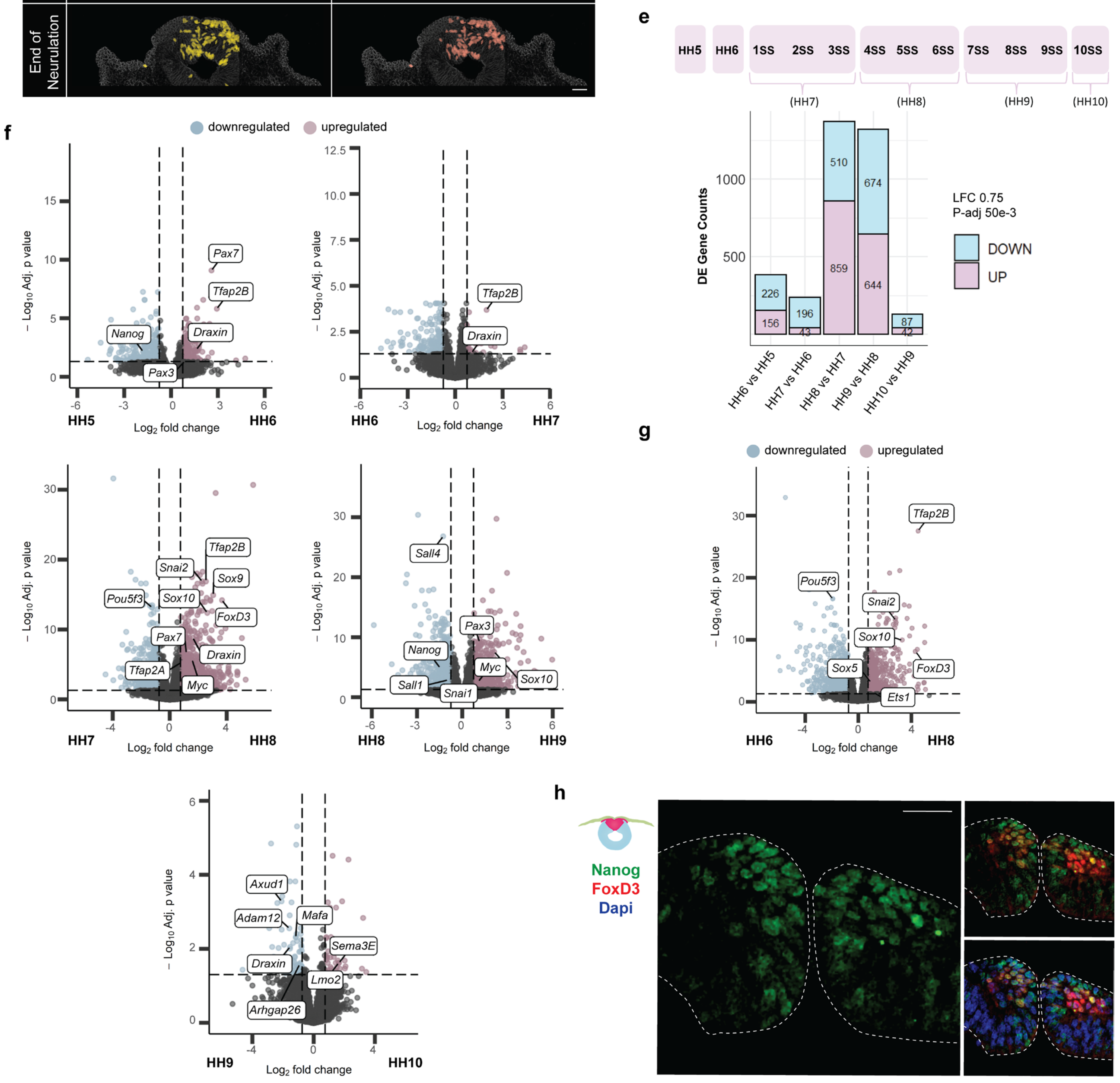

Supplementary Figure 4 | Supporting scMST, bulkRNAseq and immunofluorescence data for maintenance of pluripotency signature and existence of pan-ectodermal stem cells in the developing ectoderm.

a, A second embryo showing pseudo-colored cells with a pluripotency signature spanning the whole ectoderm until they are restricted to the dorsal neural tube at the end of neurulation recapitulates the findings and spatial patterning shown in Fig 2a. **b**, The pan-ectodermal stem cells pseudo-colored in the second embryo, which recapitulates the findings in Fig 2b. N=Notochord (midline). **c**, Table showing the number of cells that belong to the respective groups (cells with pluripotency-signature, and pan-ectodermal stem cells) represented in a and b (see also Fig. 2a-b) as well as the percentages of overlapping cells. **d**, Table showing the number of cells that belong to both of the stem cell groups from the heatmaps (transitioning and undecided from Fig. 1, Supplementary Fig. 2-3) and the pan-ectodermal stem cell group (b and Fig. 2b). The number of overlapping cells between these groups is presented together with the corresponding percentages. **e**, Numbers of differentially expressed genes from stage to stage during neural crest development analyzed from the bulk RNAseq data that was collected from the respective developing neural crest domains from each stage. The highest increase in the number of upregulated genes is between stages HH7 and HH8. **f**, Volcano plot of the Wald test always comparing two consecutive stages from the bulk RNAseq data set in d. Significantly upregulated (red) and downregulated (blue) genes are colored (p-adj <0.05, LFC 0.75) and NC and pluripotency markers are highlighted. **g**, Volcano plot of the Wald test comparing HH8 to HH6. Significantly upregulated (red) and downregulated (blue) genes are colored (p-adj <0.05, LFC 0.75) and NC markers are highlighted. **h**, Immunostaining of Nanog protein (green) in the NC domain in the dorsal neural tube together with the NC specification marker FoxD3 (red) at the end of neurulation at the midbrain level (stage HH9) demonstrates that Nanog is translated into protein at the end of neurulation in the neural crest stem cell niche domain. Scale bar = 30 μm.

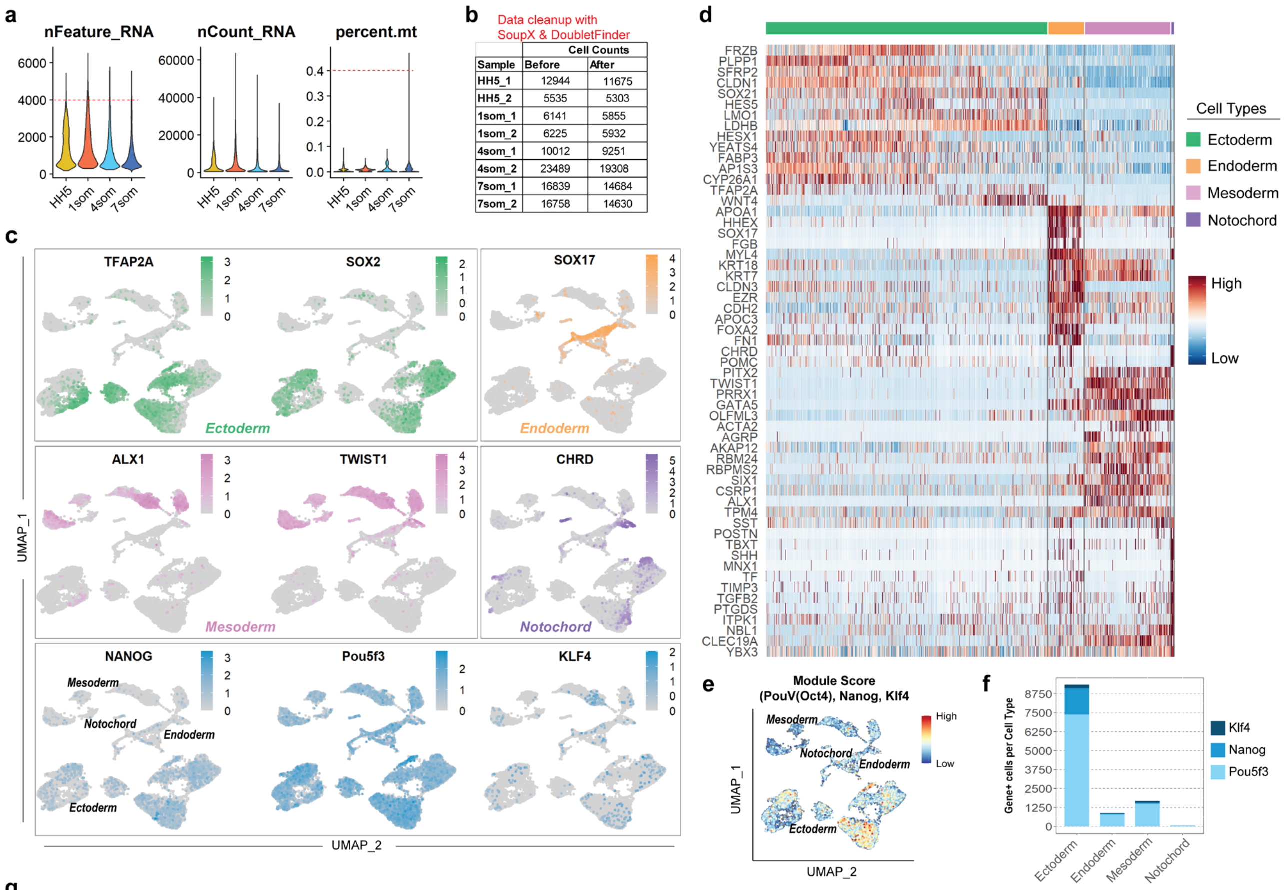

	Cell Counts	
Sample	Before	After
HH5_1	12944	11675
HH5_2	5535	5303
1som_1	6141	5855
1som_2	6225	5932
4som_1	10012	9251
4som_2	23489	19308
7som_1	16839	14684
7som_2	16758	14630

g

Stages	Total cell count per stage	cells with high PE SCs score	cells with high N score	cells with high N + PE SCs	percentage	cells with high NC score	cells with high NC + PE SCs	percentage
HH5	4595	2369	757	357	% 47.2	2	1	% 50.0
1 somite	2958	1182	581	214	% 36.8	3	3	% 100.0
4 somite	4559	1887	779	249	% 32.0	106	85	% 80.0
7 somite	3578	1557	1047	314	%30.0	372	281	%75.5

Supplementary Figure 5 | Supporting scRNAseq data.

a, Violin plots show RNA features, counts and mitochondrial ratio per each developmental stage. Dashed red line indicates threshold for filtering cells to exclude the ones with >4000 features and >0.4% mitochondrial content. **b**, Table representing cell counts in each replicate before and after cleaning the data from ambient RNA and doublets (SoupX and DoubletFinder). **c**, Feature plots show cells with high expression of known markers reflecting the three germ layers and notochord, as well as the pluripotency genes *Nanog, PouV/Oct4 and Klf4*, which are mostly found in the ectoderm. **d**, Heatmap showing top 15 differentially expressed genes in each cluster. **e**, Module score reflecting a pluripotency signature demonstrates that the cells with the highest score are found in the ectoderm. **f**, Bar Graphs highlight that ectoderm contains most of the cells that express pluripotency genes, as demonstrated by using the pooled scRNaseq data from two replicates per stage (positive counts for the three pluripotency genes individually in each cluster). **g**, Table showing the numbers of cells for module scoring in Fig 3i. Note that the expression of genes of the neural crest score only emerge at late neural fold stage. PE SC = Pan-ectodermal stem cells.

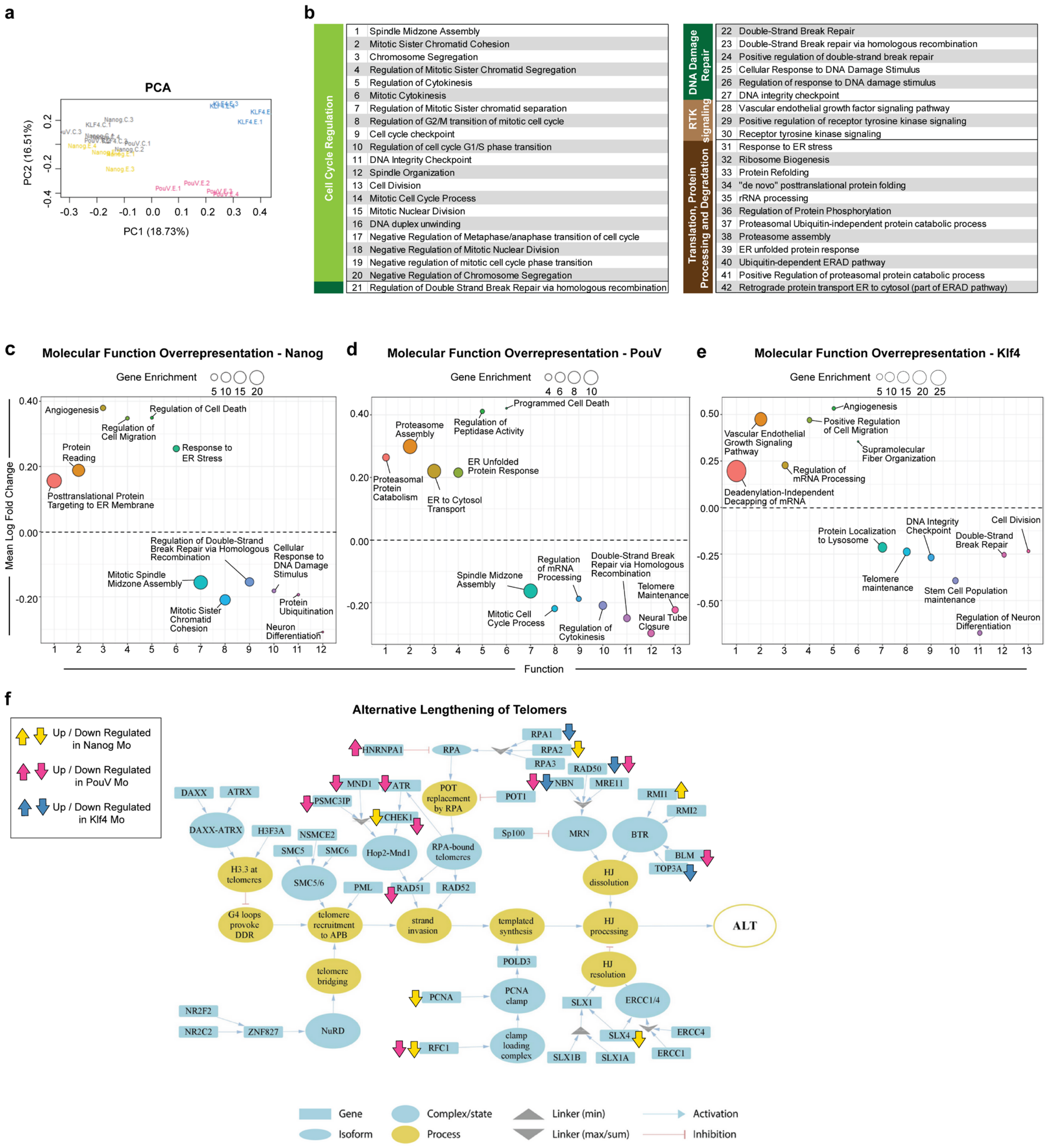

Supplementary Figure 6 | Supporting bulk RNAseq analysis data on individual knockdowns of pluripotency genes.

a, Separation of bulk RNAseq samples in Principal Component Analysis. Samples of each knockdown cluster separately, whereas the control sides of all experiments cluster together. $n = 4$ biological replicates for each experimental samples and $n = 9$ biological replicates for control samples, each sample consists of cranial neural plate border regions from HH7-8. **b**, List of differentially expressed cellular functions contributing to cell cycle regulation, DNA repair, RTK-signaling and protein processing in Fig 5c. **c-e**, Molecular Function Overrepresentation were obtained

by using PANTHER (GO biological process). Fold enrichments were obtained using statistical overrepresentation test, and p-values were calculated using Fisher's Exact test and adjusted using the Bejamini-Hochberg false discovery rate (FDR) method for multiple test correction. The plots reflecting the differentially expressed genes of the respective knockdown of **c**, Nanog, **d**, PouV/Oct4 and **e**, Klf4. **f**, A schematic representation of alternative lengthening of telomeres pathway (http://big.sci.am/software/tmm/) highlights how different components of the same pathway are affected by the respective knockdowns of Nanog, PouV/Oct4 and Klf4.

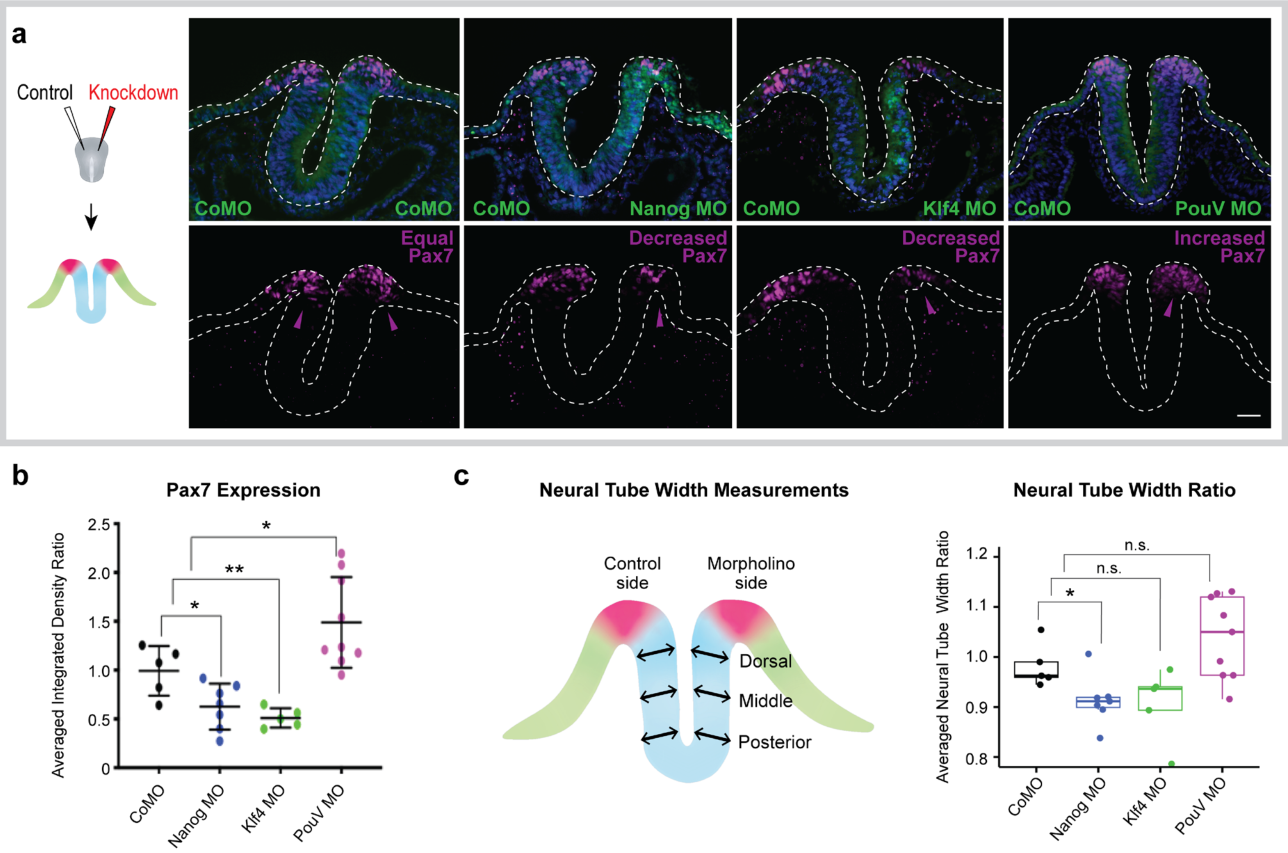

Supplementary Figure 7 | Individual morpholino-mediated knockdowns of the pluripotency genes shows different impact on neural crest development.

a, Individual knockdowns of pluripotency genes affect Pax7 expression in the neural plate border. Scale bar = 30 µm. **b**, Integrated Density of Pax7 expression was measured for each side. The ratio of the experimental morpholino side to its contralateral control morpholino side was calculated, based on an average value of at least seven sections per embryo (n = 1). The obtained ratio was then compared to that of control embryos injected with a control morpholino on both sides. To determine the significance of the difference between experimental (Nanog MO, n = 6 embryos, Klf4 MO n = 5 embryos, PouV MO n = 9 embryos) and control (CoMO n = 5 embryos) ratios, unpaired t-test was applied (* = $p < 0.05$, ** = $p < 0.01$). **c**, A cartoon representation illustrating the regions where neural tube thickness was measured (dorsal, middle, and posterior parts of the neural tube). Measurements from these three sections were averaged per cross-section, following the same sample size (n) as in **b.** The width of the experimental morpholino side was compared to the width of the contralateral control morpholino side, and the resulting ratio was compared to the ratio from control embryos injected with a control morpholino on both sides. Statistical significance was assessed using an unpaired Student's t-test (* = $p < 0.05$). For b and c, see Supplemental Data 6 for exact p values. Source data showing data points for b and c are provided as a Source Data file.
