# Supplementary material for: Maintenance of pluripotency-like signature in the entire ectoderm leads to neural crest stem cell potential
Source: Nat Commun. 2023 Sep 23;14:5941. doi: 10.1038/s41467-023-41384-6 (PMC10518019; doi:10.1038/s41467-023-41384-6)
Supplement: Supplementary file 3 — Description of Additional Supplementary Files [file 41467_2023_41384_MOESM3_ESM.pdf]

<u>Description of Additional Supplementary Files</u>

Supplementary Data 1: Up regulated gene list and their p-adj, p-values along with LFC for time series bulk RNAseq data

Supplementary Data 2: Down regulated gene list and their p-adj, p-values along with LFC for time series bulk RNAseq data

Supplementary Data 3: Differentially expressed gene list and their p-adj, p-values along with LFC for scRNAseq data (germ layers)

Supplementary Data 4: Differentially expressed gene list and their p-adj, p-values along with LFC for scRNAseq data (Pan Ectodermal cells vs Others)

Supplementary Data 5: MO bulk RNAseq commonly differentially expressed genes among all three MO

Supplementary Data 6: Box Plot statistics for Supplementary Figure 7

Supplementary Data 7: All probe sequences used for scMST experiments.

Supplementary Data 8: key resources table
